# Ambulatory Multi-Drug Resistant Tuberculosis Treatment Outcomes in a Cohort of HIV-Infected Patients in a Slum Setting in Mumbai, India

**DOI:** 10.1371/journal.pone.0028066

**Published:** 2011-12-01

**Authors:** Petros Isaakidis, Helen S. Cox, Bhanumati Varghese, Chiara Montaldo, Esdras Da Silva, Homa Mansoor, Joanna Ladomirska, Giovanni Sotgiu, Giovanni B. Migliori, Emanuele Pontali, Peter Saranchuk, Camilla Rodrigues, Tony Reid

**Affiliations:** 1 Médecins Sans Frontières, Mumbai, India; 2 Médecins Sans Frontières, Cape Town, South Africa; 3 Monash University, Melbourne, Australia; 4 Hygiene and Preventive Medicine Institute, University of Sassari, Sassari, Italy; 5 S. Maugeri Foundation, World Health Organization Collaborating Centre for Tuberculosis and Lung Diseases, Tradate, Italy; 6 Deptartment of Infectious Diseases, Galliera Hospital, Genoa, Italy; 7 P.D. Hinduja National Hospital and Medical Research Centre (Hinduja), Mumbai, India; 8 Médecins Sans Frontières, Operational Research Unit, Brussels, Belgium; London School of Hygiene and Tropical Medicine, United Kingdom

## Abstract

**Background:**

India carries one quarter of the global burden of multi-drug resistant TB (MDR-TB) and has an estimated 2.5 million people living with HIV. Despite this reality, provision of treatment for MDR-TB is extremely limited, particularly for HIV-infected individuals. Médecins Sans Frontières (MSF) has been treating HIV-infected MDR-TB patients in Mumbai since May 2007. This is the first report of treatment outcomes among HIV-infected MDR-TB patients in India.

**Methods:**

HIV-infected patients with suspected MDR-TB were referred to the MSF-clinic by public Antiretroviral Therapy (ART) Centers or by a network of community non-governmental organizations. Patients were initiated on either empiric or individualized second-line TB-treatment as per WHO recommendations. MDR-TB treatment was given on an ambulatory basis and under directly observed therapy using a decentralized network of providers. Patients not already receiving ART were started on treatment within two months of initiating MDR-TB treatment.

**Results:**

Between May 2007 and May 2011, 71 HIV-infected patients were suspected to have MDR-TB, and 58 were initiated on treatment. MDR-TB was confirmed in 45 (78%), of which 18 (40%) were resistant to ofloxacin. Final treatment outcomes were available for 23 patients; 11 (48%) were successfully treated, 4 (17%) died, 6 (26%) defaulted, and 2 (9%) failed treatment. Overall, among 58 patients on treatment, 13 (22%) were successfully treated, 13 (22%) died, 7 (12%) defaulted, two (3%) failed treatment, and 23 (40%) were alive and still on treatment at the end of the observation period. Twenty-six patients (45%) experienced moderate to severe adverse events, requiring modification of the regimen in 12 (20%). Overall, 20 (28%) of the 71 patients with MDR-TB died, including 7 not initiated on treatment.

**Conclusions:**

Despite high fluoroquinolone resistance and extensive prior second-line treatment, encouraging results are being achieved in an ambulatory MDR-T- program in a slum setting in India. Rapid scale-up of both ART and second-line treatment for MDR-TB is needed to ensure survival of co-infected patients and mitigate this growing epidemic.

## Introduction

In India, multidrug-resistant tuberculosis (MDR-TB) is estimated to account for 2.3% (95% Confidence Intervals (CI): 1.8–2.8) of new cases and 17.2% (95% CI: 14.9–19.5) of previously treated TB cases [1]. Reports from several states suggest that the prevalence of MDR-TB among previously-treated patients varies around the country, with values ranging from 7% to over 50% [2]–[5]. A relatively recent publication from Mumbai showed a high MDR-TB prevalence of 24% and 41% among new and re-treatment cases respectively [6]. WHO estimate the number of MDR-TB cases in 2009 in India at 99,000 (79,000–120,000) which represents approximately 25% of the global burden of MDR-TB [1].

Infection with Human Immunodeficiency Virus (HIV) further complicates the management of MDR-TB. India has the third highest HIV burden in the world with an estimated 2.5 million people living with HIV/AIDS in 2006 [7]. Data from other settings suggest that mortality from MDR-TB in HIV-infected patients is extraordinarily high, particularly within the first 30 days after diagnosis [8].

Systematic reviews have shown that few studies describe MDR-TB treatment outcomes from high HIV prevalence settings and that HIV status is often inconsistently reported [9], [10]. Although the use of antiretroviral therapy (ART) with MDR-TB treatment has been shown to improve outcomes in co-infected patients [9], [10], [11], treatment of MDR-TB in HIV-infected patients remains a challenge. Patients are required to take large numbers of pills each day and there is the potential for additive side effects and drug interactions between antiretroviral agents and second-line anti-tuberculosis drugs [12].

Overall, there is a serious deficiency in reports describing treatment of MDR-TB in HIV-infected patients, especially in programmatic settings in resource-constrained countries. A recent study from Lesotho showed that starting early empirical treatment for suspected MDR-TB patients using a community-based treatment approach in mainly HIV-infected adults resulted in high culture conversion, although significant mortality remained [13].

While Mumbai has a widely accessible DOTS program in place under the Revised National Tuberculosis Control Program (RNTCP) for treatment of drug-susceptible tuberculosis, treatment for drug-resistant TB was, until recently, only available through the private sector. As a result, MDR-TB treatment has often been administered in a chaotic, unregulated manner using non-pre-qualified anti-TB drugs and at prohibitive prices for the large majority of patients. Given the large MDR-TB disease burden, there is an urgent need to scale up appropriate treatment for MDR-TB in India and in order to achieve this, a decentralized approach may be most appropriate. Special focus should be on early diagnosis and treatment initiation, particularly among HIV-infected populations.

In February 2006, Médecins Sans Frontières (MSF) started an HIV project in Mumbai, offering care and treatment to people living with HIV unable to access the public system or who could not obtain appropriate ART regimen, including second-line antiretroviral drugs. MSF has been treating MDR-TB among HIV-infected individuals in this project since May 2007. MDR-TB treatment subsequently became available in the public sector in Mumbai in late 2010.

This report aims to describe patient and treatment outcomes in a cohort of HIV/MDR-TB co-infected patients treated on an outpatient basis, in an urban, overpopulated slum setting in Mumbai, India. To our knowledge, this is the first analysis of MDR-TB patient outcomes in HIV-infected patients in India.

## Methods

### Study design

This was a prospective, observational cohort study using data routinely collected at each consultation and entered into an electronic database.

### Setting and study population

MSF has been operating a clinic in Khar, a suburb in Mumbai, India since 2006. It is specialized in HIV care and provides treatment free of charge to patients referred by accredited public and public-private Antiretroviral Treatment Centers (ART Centers) from the greater Mumbai area and by a network of community non-governmental organizations (NGOs). The great majority of patients are slum-dwellers.

An MDR-TB component was added to the HIV treatment program in May 2007. MDR-TB care and treatment is offered on an outpatient-clinic basis. In general, patients have been referred to the clinic for evaluation of confirmed or suspected MDR-TB, by clinicians working in the Ministry of Health and Family Welfare network of ART centers, and by private physicians or community NGOs.

All HIV-infected patients with bacteriologically confirmed MDR-TB or those suspected to have MDR-TB, based on clinical findings and TB treatment history but without bacteriological confirmation who were followed up in the clinic between May 2007 and May 2011 were included in this study.

### Mycobacterial Culture and Drug Susceptibility Testing (DST)

HIV-infected patients with symptoms suggestive of TB or any patient referred to the clinic with MDR-TB suspicion were screened with two sputum samples for acid-fast bacilli (AFB) by sputum smear microscopy and two for AFB culture. Culture and DST was performed using BACTEC MGIT 960 system (Becton-Dickson, Sparks, Maryland, USA). Drug susceptibility testing (DST) was performed on all culture positive isolates against four first-line TB drugs: isoniazid, rifampicin, ethambutol and streptomycin and four second-line drugs (kanamycin, ethionamide, para-aminosalicylic acid (PAS) and ofloxacin) using World Health Organization (WHO) approved guidelines for drug concentrations. Samples were processed in the Laboratory Medicine Department of the P. D. Hinduja National Hospital & Medical Research Centre, Mumbai, which is accredited by the Revised National Tuberculosis Control Program (RNTCP) and the College of American Pathologists (CAP) to perform first line DST to Streptomycin, isoniazid, rifampicin and ethambutol. For the phenotypic second line DST, the laboratory was performing internal quality control weekly and twice a year inter –laboratory external quality control with CAP accredited laboratories. Some patients (approximately ten) brought DST results from various private TB laboratories in Mumbai showing resistance to isoniazid and rifampicin. Patients were started on treatment with second-line TB drugs, after baseline investigations and counselling were completed, and were asked to produce sputum for culture and DST before starting treatment.

### Treatment protocol

The clinic treatment protocols follow WHO international guidelines [12]. Whenever possible, an individualized treatment regimen was designed for each patient, based on the first and second line DST results and on patient's treatment history. A standardized treatment regimen was used for empiric treatment in those patients who required prompt treatment initiation due to the severity of their disease.

The standardized regimen included six drugs: pyrazinamide, capreomycin, moxifloxacin, ethionamide, cycloserine and PAS. The standardized regimen was modified once the DST results became available. The same standardized regimen was continued for patients with unconfirmed MDR-TB, diagnosed on clinical grounds and treatment history. All TB drugs were dosed according to bodyweight. Dosing and drugs were also changed in response to severe adverse effects. The severity of adverse events was defined by laboratory criteria (whenever quantifiable) or based on effect on patient tolerance and adherence. Adverse events were aggressively managed and regimen modification was done as last resort.

Patients, unless already on ART, were started on antiretroviral drugs as soon as they were tolerating second-line TB drugs, irrespective of CD4 cell count. Two nucleoside reverse transcriptase inhibitors and one non-nucleoside reverse transcriptase inhibitor were used for patients being prescribed 1^st^ line ART, while patients in need of 2^nd^ line ART (mostly patients with virological failure, defined as two consecutive, detectable HIV RNA viral loads) received a protease inhibitor-based regimen [14].

### Treatment initiation and follow-up

Patients in stable clinical condition were started on MDR-TB treatment on an ambulatory basis. Patients were evaluated by a multidisciplinary team of trained physicians, nurses, social workers and psychologists. The same team provided care and treatment for HIV in a “one stop” service. The attending physician examined patients clinically at least once a week in the first month of treatment and once or twice a month thereafter, depending on the patient's clinical condition. Special attention was given to identify gastro-intestinal adverse events, hearing loss or tinnitus (including monitoring audiograms), depressive or psychotic symptoms, and signs of hypothyroidism. Sputum smear and culture were repeated monthly until the end of the intensive treatment phase and every other month during the continuation phase. Chest X-ray was not routinely used for treatment monitoring.

In the very early stages of the program, a self-administered treatment strategy was tried, but the strategy soon shifted to twice-daily directly observed treatment (DOT). DOT was planned to be available no more than 10 minutes walking distance from patients' homes. This was possible thanks to a community network of public/private health structures and non-governmental organizations (NGOs) that acted as DOT providers. These included public health posts, private practitioners and local NGOs, the great majority of which were based in slums.

DOT providers were trained to provide DOT, administer injections and monitor for adverse effects. MSF supplied the providers with second line TB drugs, materials to give injections, masks and N95 respirators, and training on infection control. Each DOT provider was supervised weekly by phone and monthly by an MSF staff visit. Patients went to the DOT provider daily to receive TB treatment and monthly to the MSF clinic for medical and psychosocial follow-up.

Patients who were unable to ambulate or were otherwise unstable clinically were admitted to a charity hospital under the supervision of the MSF clinical team. Patients were also admitted if they experienced severe adverse effects or other clinical complications precluding outpatient management. Treatment was continued for a minimum of 18 months total duration, including a minimum of 6 months intensive treatment with the injectable agent. All treatment was provided free of charge.

### Data collection

Demographic and clinical information were systematically recorded on standardized clinical files designed specifically for the program. Information on all patients was prospectively collected and entered into an electronic database. Information on HIV and antiretroviral treatment was collected in the same patient file but entered in a separate database. Each patient had a unique identification code that was used in both databases. Trained personnel extracted clinical, treatment, and laboratory data from individual patient records regularly and entered them into both databases. A full time data manager routinely checked data entry for accuracy and completeness.

### Ethics

The study protocol was approved by the Independent Ethics Review Board of Médecins Sans Frontières, Geneva, Switzerland. As this was a study of routinely collected monitoring data, informed consent from the patients was not obtained. The named ethics committee specifically waived the need for consent.

### Statistical methods

Data from all patients diagnosed with MDR-TB between May 2007 and May 2011 were used in the analyses. Patient characteristics at admission in the MDR-TB program and their status at the end of the study period were summarized using descriptive statistics. Kaplan-Meier analysis was performed to assess survival probability. Patient follow-up ended when an outcome was recorded, or censored on May 15, 2011. Possible outcomes, defined according to WHO guidelines, included: cure, treatment completed, death, default, transfer out, treatment failure, and ‘alive and on treatment’ [12], [15]. Microsoft Excel and SPSS (version 16.0, Chicago, IL) were used for analysis.

## Results

### Patient characteristics

**Figure 1:**
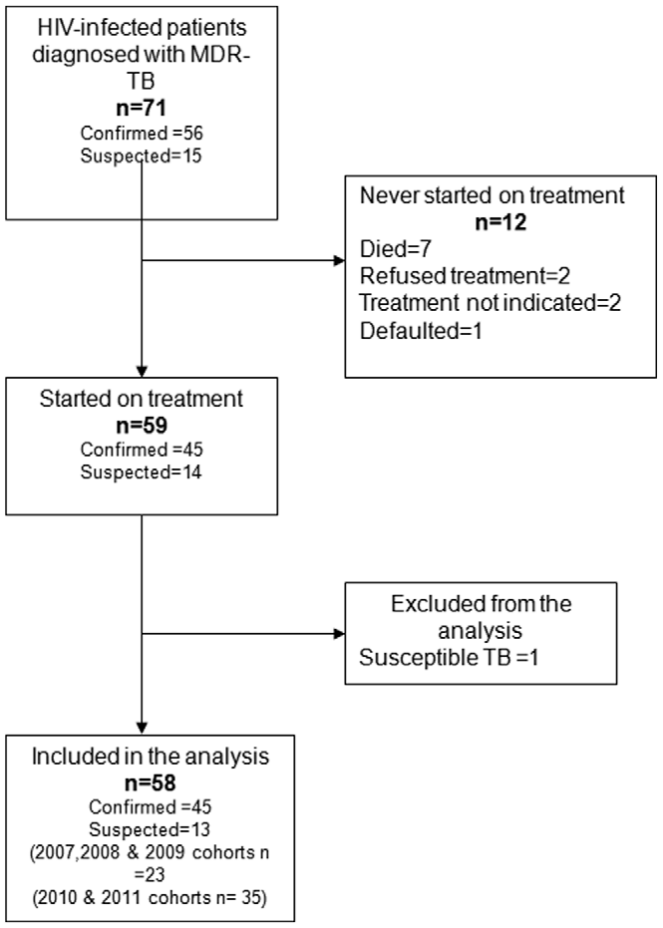
Flowchart of patients with MDR-TB/HIV co-infection enrolled in care between 2006 and 2011, Mumbai, India.

Between May 2007 and April 2011, 71 patients were diagnosed with MDR-TB (57 (80%) bacteriologically confirmed and 14 (20%) unconfirmed) and registered in the MSF clinic (Figure 1). Twelve of these patients never started MDR-TB treatment: seven of them died before treatment initiation, two patients refused treatment after counselling, one patient was lost to follow-up and for two patients second-line anti-TB treatment was not considered medically indicated: one patient dramatically improved on first-line anti-TB treatment while waiting for the DST results (including culture conversion) and for the second patient there were no therapeutic options available other than palliative care (patient susceptible only to PAS). All of the remaining 59 patients started treatment with second-line TB drugs. One of the suspected patients was subsequently found to be infected with pan-susceptible *M. tuberculosis* strains after three months of second-line treatment. This patient was switched to first-line treatment and excluded from the outcome analysis (Figure 1). Baseline characteristics of the patients are shown in Table 1.

**Table 1:** Baseline characteristics of HIV-infected MDR-TB patients enrolled in care.

	Started on MDR-TB treatment	Total
Number of patients enrolled	59	71
Confirmed	45	57
Unconfirmed	14	14
**Demographics**		
Age in years, median (min-max)	35 (11–61)	35 (11–61)
Women, n (%)	26 (44)	35 (49)
Social Stratum n (%)		
Lower	42 (71.2)	46 (64.8)
Middle	11 (18.6)	12 (16.9)
Upper	2 (3.4)	2 (2.9)
Unknown	4 (6.8)	11 (15.4)

TB History & clinical presentation	Co-infected patients who started MDR-TB treatment (n = 58)
Previous TB treatment n (%)	
Public sector	26 (44.8)
Private sector	9 (15.5)
Both sectors	16 (27.6)
No previous treatment	5 (8.6)
Unknown	2 (3.4)
Exposed to 2nd line anti TB drugs	
Yes	26 (44.8)
NO	28 (48.3)
Unknown	4 (6.9)
Sputum or other sample smear at treatment initiation n (%)	
Positive	25 (43.1)
Negative	33 (56.9)
Sputum or other sample culture at treatment initiation n (%)	
Positive	47 (81)
Pulmonary/Extra pulmonary	38/9
Negative	11 (20.7)
Pulmonary/Extra pulmonary	8/3
Tuberculosis site n (%)	
Pulmonary	50 (86.2)
confirmed/suspected	39/11
Extra pulmonary	8 (13.8)
confirmed/suspected	5/3

HIV History & clinical presentation	Co-infected patients who started MDR-TB treatment (n = 58)
Median (IQR) CD4-count at MDR-TB treatment initiation (cells/µl)	135 (85–193)
ART treatment history n (%)	
On ART before anti-MDR-TB	37 (63.8)
On ART after anti-MDR-TB	18 (31)
No ART	3 (5.2)
Median time from MDR-TB treatment start to ART initiation : months (IQR)	1.1 (1.1–4.7)
ART n (%)	
First line	45 (77.6)
Second line	8 (13.8)

### Tuberculosis clinical characteristics and treatment history

Twenty-five (43%) of the 58 patients were sputum AFB-positive and 47(81%) were culture positive before starting treatment. Ten patients were smear- and culture-negative, four of whom had extra-pulmonary TB. Overall, 50 patients (86%) had pulmonary TB, seven of whom were diagnosed with both pulmonary and extra-pulmonary TB (three abdominal, three cerebral and one vertebral TB). Eight patients (14%) were diagnosed as having only extra-pulmonary TB, three of whom had signs of TB at more than one site. Among the patients with pulmonary TB, 39 were confirmed and 11 remained unconfirmed, while 5 extra pulmonary TB patients were bacteriologically confirmed and three were started on treatment based on clinical condition and TB treatment history (Table 1).

All but five patients (91%) had received previous TB treatment, half of them in the public sector and half of them in the private or both sectors. Strikingly, half of these patients had a history of previous exposure to second-line TB drugs, most commonly fluoroquinolones. This is reflected in the 40% ofloxacin resistance among bacteriologically confirmed cases in this patient cohort (Table 2). No MDR-TB patients were diagnosed with resistance to second-line injectable drugs. Three patients (6% among bacteriologically confirmed) were diagnosed with extensively drug-resistant TB (XDR-TB).

**Table 2:** Resistance patterns in a Mumbai cohort of HIV/MDR-TB co-infected patients.

Drug resistance profile	No previous TB treatment or first line treatment only	Previously exposed to second line drugs
Suspected MDR-TB	6	7
MDR, but no second line resistance		
HR	1	1
HR+other first-line	8	7
MDR with second line resistance (but not XDR)		
HR+Ofx	7	11
HR+second-line injectable	0	0
HR+Group 4 drugs	6+	2
XDR	2	0

+ including one strain with isoniazid susceptibility.

R: Rifampicin, H: Isoniazid, Ofx: ofloxacin.

### HIV clinical characteristics and treatment history

The median CD4-count at the time of MDR-TB treatment initiation was 135 cells/µl (IQR: 85–193). Most patients, especially during the later years, were registered in the clinic at the time of diagnosis of MDR-TB. Thirty-seven patients (64%) were on ART before a diagnosis of MDR-TB was made. Eight patients were on second line ART. Three patients never started ART: two migrant workers from Nepal who defaulted early in MDR-TB treatment and one patient who was about to start at the time of the analysis.

### Treatment outcomes

Among the 23 patients initiated on treatment prior to May 2009 (24 months of follow-up), seven patients (31%) were cured, four (17%) have completed treatment, four patients (17%) died, six defaulted (26%) and two patients (9%) failed treatment.

Among these 23 patients, eight TB strains were resistant to ofloxacin ; two of them completed treatment, one died and five defaulted. Patients without fluoquinolone resistance (15) were more likely to be cured and less likely to default than patients with resistance to fluoquinolones (p<0.05). The two patients who failed treatment had no baseline resistance to ofloxacin or to injectable drugs.

Overall among the total 58 patients, eight patients (14%) were cured, five (9%) completed treatment, 13 patients (22%) died, seven defaulted (12%), two patients (3%) failed treatment and 23 patients (40%) were alive and in treatment at the end of the observation period.

Out of 25 patients with a positive sputum smear at baseline, thirteen (52%) achieved sputum smear conversion in the first two to four months of treatment (median 2 months). Similar rates of culture conversion were observed. Out of the 44 patients with a baseline positive culture, 23 (52%) achieved culture conversion in the first four months of treatment.

### Mortality, causes of death and loss to follow-up

**Figure 2:**
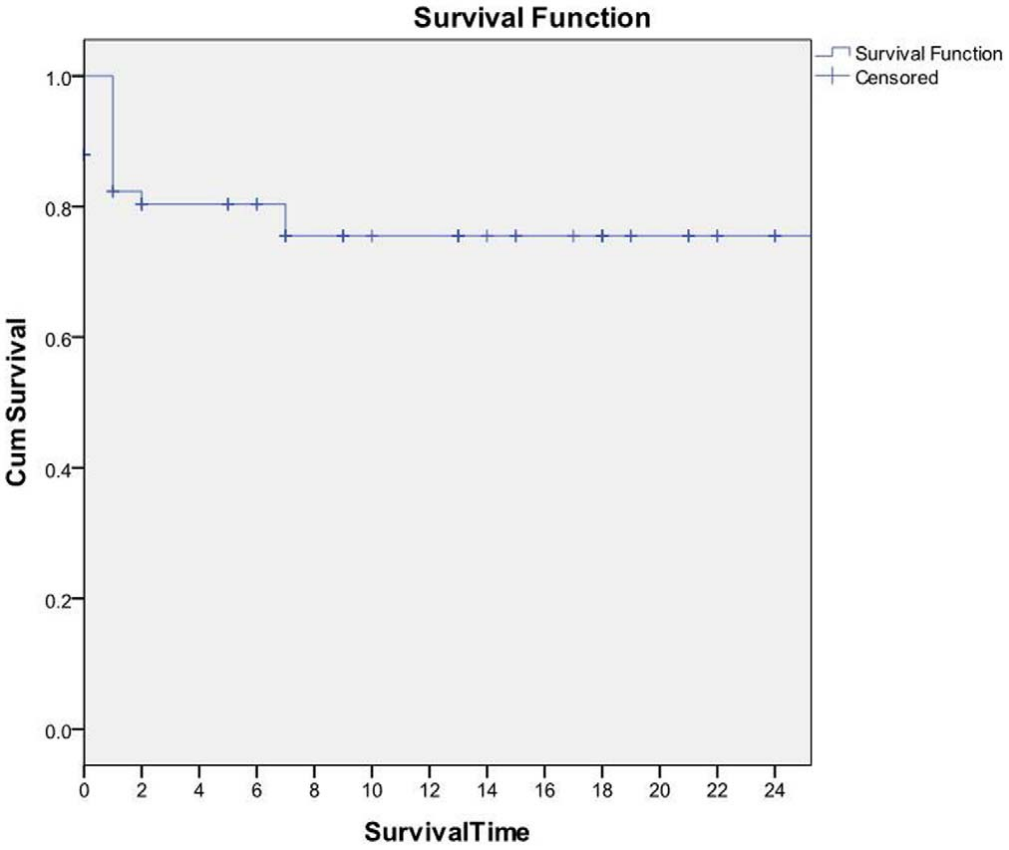
Time to death under MDR-TB treatment.

Survival curves for the 58 patients who started on treatment are shown in Figures 2a and 2b. Survival is measured in months after starting MDR-TB treatment. Crosses indicate censored patients. The probability of survival at 24 months of treatment was 0.75.

Death occurred after a median of 27 days in treatment (range 1–229 days). Causes of death for the 13 patients who died included four deaths not related to TB or treatment (one head injury, one renal failure, one diabetic ketosis and one HIV wasting syndrome).

Among the seven patients who did not complete treatment, one was a Nepalese migrant worker who decided to repatriate, one a sex worker who had problems to access the clinic and one a 16 year old adolescent who refused to continue after 15 months of treatment despite the absence of adverse events and receiving intensive counseling and psychosocial support. Two patients diagnosed with XDR-TB were also lost to follow-up despite efforts from the psychosocial team to trace them. Two more patients had no apparent reason to default.

### Adverse events

Adverse events associated with second line DR-TB treatment are summarized in Table 3.

**Table 3:** Adverse drug reactions in MDR-TB/HIV co-infected patients under treatment.

	Reactions n (%)	Regimen change (n)
Hypothyroidism/Altered thyroid function tests	19 (33)	0
Hypokalemia	8 (14)	0
Gastrointestinal symptoms	7 (12)	0
Psychosis	7 (12)	3
Loss of hearing	5 (9)	4
Tendonitis	3 (5)	3
Renal impairment	2 (3)	0
Depression	2 (3)	1
Peripheral Neuropathy	2 (3)	0
Arthralgia	2 (3)	0
Abscess at injection site	2 (3)	0
Seizures	1 (2)	1
Vertigo/ataxia	1 (2)	0

The most common adverse drug reactions were related to abnormal thyroid function (assessed by TSH, T3 and T4 measurements) due to PAS and/or ethionamide, and hypokalaemia, due to the aminoglycosides and capreomycin. Drug dosages had to be adjusted in two patients with renal impairment. For patients with psychosis/depression, seizures, hearing loss and tendonitis, the drugs assumed to be responsible were discontinued. All other events were managed symptomatically and without physician-directed discontinuation of therapy.

### Immunological recovery

**Figure 3:**
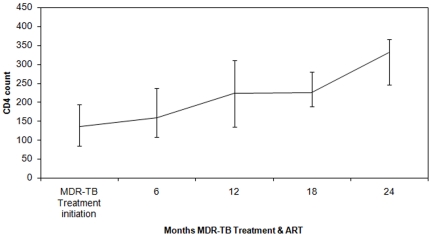
CD4 evolution over time in MDR-TB/HIV co-infected patients under ART and MDR-TB treatment, Mumbai, India.

Out of 58 patients who started TB treatment, 37 were already on ART, 18 started ART during TB treatment and three never started as previously described. Figure 3 shows the CD4-count evolution over time, under MDR-TB treatment and ART: the median CD4-count increased to 224 cells/µl (IQR: 135–311) and 331 cells/µl (IQR: 247–367) after one and two years of treatment respectively.

## Discussion

This is, to our knowledge, the first report on MDR-TB treatment outcomes from a cohort of HIV-infected patients in India [9], [10]. Despite India's large burden of MDR-TB there are discouragingly few reports of successful treatment programs from the sub-continent [16]. A recent study from the Tuberculosis Research Center in Chennai, reported good MDR-TB treatment outcomes in a cohort of 38 patients, however, HIV-infected patients were excluded from the study [17]. Our study of a Mumbai cohort of HIV-infected patients only, shows encouraging results in terms of survival, cure rates, culture conversion and immunological recovery, especially considering high rates of fluoroquinolone resistance and previous second-line treatment in this cohort. MDR-TB treatment programs with largely HIV-negative patients have reported cure rates and death rates of 61–77% and 5–19% respectively [9], [10], [18]–[21]. The early results of a treatment program in Lesotho [13], however, showed that MDR-TB treatment outcomes in HIV-infected cohorts were likely to be significantly worse: overall, 29% of patients died. There was a trend towards poorer outcomes in HIV-infected individuals, with significant mortality in the first few weeks after DR-TB treatment initiation; these results were consistent with previous studies showing increased mortality in HIV-positive compared to HIV-negative patients with drug-susceptible TB [22], [23]. We observed lower mortality rates in our cohort of co-infected patients; however, the high defaulter rate recorded may mask unreported mortality. The high early mortality seen in the cohorts of HIV-infected patients argues strongly for early initiation of both ART and second-line TB drugs.

In Mumbai, MDR-TB treatment was until recently (July 2010) only available through an unregulated, private health sector [16]. However, the private sector does not generally consider the risk of resistance amplification or the ongoing challenges of adherence to a very long and costly treatment regimen fraught with a high pill burden and significant adverse events. Private physicians rarely (if ever) use DOT as treatment strategy. The particular setting of Mumbai, a mega-metropolis where the marginalised may have inadequate nutrition, rudimentary sanitation and poor home-ventilation is particularly challenging. Difficult transportation and an extremely mobile population, further complicates the management and the follow-up of these patients. Thirty-seven percent of the patients in our cohort had already received second-line TB treatment before they were enrolled in the MSF clinic. According to their medical records and referral notes (when available), they were often on erratic regimes, under-dosed and had their treatment interrupted as they could not afford the high cost of second line anti-TB drugs for long periods of time [24], [25].

The Mumbai MSF clinic has been trying to address these MDR-TB challenges since the program started in 2007. The outcomes recorded so far in the program demonstrate the feasibility of treating HIV/MDR-TB co-infected patients in such a setting. Given the high rate of resistance to fluoroquinolones in India [4], [26] and erratic previous treatment for many of the patients the outcomes are reasonable. We suggest that the following factors contributed to these results: first, all patients received DOT thanks to a network of individual DOT providers, allowing each patient to easily access care within walking distance from his/her home. By moving treatment into the community, it was possible to overcome problems of patient access to clinics. Second, the monitoring plan for early identification of adverse events, that included systematic clinical and laboratory assessment, and the availability of ancillary drugs helped to promptly diagnose and appropriately manage adverse drug effects. Third, therapy followed internationally recommended guidelines with maximally effective drug combinations, based on DST, and at the highest recommended doses [12]; this likely reduced the risk of treatment failure and amplification of resistance. Fourth, psychosocial support was given to all patients with monthly home visits and specific support tools were developed. A multidisciplinary team (doctors, nurses, counsellors, psychologists) was essential to manage adherence challenges and psychological issues related to the disease and its treatment, stigma and isolation. Fifth, TB and HIV care were fully integrated: patients received treatment for both conditions in the same clinic as part of a “one-stop service”. This increased convenience for patients, saved time and travel expenses, and facilitated detection of drug interactions and adverse events. Moreover, the integrated approach has broadened expertise in the clinic team on the management of co-infected patients. Sixth, ambulatory treatment and follow-up may have reduced default and the risk of re-infection during hospitalization [27].

A large investment has been made in the clinic in TB infection control measures that prevent transmission of TB. A comprehensive infection control plan was designed and implemented, including environmental and administrative measures and personal protective equipment for the clinic staff. The clinic defined specific MDR-TB days, carefully organized the spacing between MDR-TB consultations, improved ventilation in consultations rooms, corridors and waiting areas and trained the staff in prevention of TB transmission. An infection control committee was established and it is now planned to expand infection control measures to the community and household level through innovative, small-scale interventions.

However, several challenges remain in this program. The high pre-treatment and early mortality rates highlight the need for intensive case finding strategies, rapid diagnosis and initiation of DR-TB treatment to reduce mortality and improve outcomes. Late referral to the clinic and advanced disease may partly explain these high rates. Studies from HIV programs have shown than even if excellent outcomes can be achieved and maintained among patients receiving antiretroviral therapy, program outcomes such as high pre-treatment mortality and pre-treatment loss-to-follow-up rates remain largely unreported [28], [29]. Similarly, failure to initiate therapy and default from treatment rates were high in this cohort, despite the provision of counselling and other psychosocial support. Our data suggests that default from treatment may be lower in more recent cohorts. While the MSF clinic reports very low loss-to-follow-up rates among non-MDR-TB patients on ART (<3%) it is disappointing that the overall rate was 26% for the co-infected patients on treatment. While there are known challenges related to the management of co-infection, several measures may have to be urgently taken to analyze and address this important issue. In our clinic, for example, we identified important gaps in patient support tools and interventions, including lack of adequate emotional and psychosocial support as well as limited access to mental health services. We have also noticed that patients referred to our clinic from the private sector have already been on various treatment regimens for long time periods and often experience treatment fatigue. Support from families is also reported to wane over time.

In this Mumbai cohort, several patients were started empirically on second-line drugs before DST results were available, based on WHO recommendations [30]. The RNTCP in India is particularly concerned with empirical treatment and strongly discourages clinicians to initiate treatment without laboratory confirmation. Considering the challenges with the unregulated private sector and the lack of trained and experienced clinicians in the management of MDR-TB in the Indian public sector, these concerns may be well justified. Ideally rather than relying on empiric treatment, efforts should be directed at introducing more rapid diagnosis that will offer both bacteriological confirmation and result in more rapid treatment initiation. The recently approved Xpert MTB/RIF (Xpert) test, has been demonstrated to be feasible in different high burden settings and has the potential to dramatically improve case detection for drug-resistant TB [31], [32]. Moreover, a recent study suggested that Xpert MTB/RIF shows good potential in the diagnosis of extrapulmonary TB and this is relevant for HIV-infected individuals [33].

In Mumbai, a community-based model of MDR-TB treatment was found to be feasible, as has been shown in other countries [13], [18], [27], [34]–[36]. DOT providers at the community level were trained to provide injections, observe pill-taking, monitor for adverse effects and support and refer patients. Adverse events occurred relatively often in this cohort, similar to a cohort from Lesotho [13]. We recorded relatively high rates of hypothyroidism and psychosis. However, with aggressive management of side effects, alterations to the TB regimen were rarely necessary.

There are some limitations to our analysis. Overall, the size of the cohort is relatively small and therefore does not allow definitive conclusions to be drawn regarding factors that may contribute to patient outcomes, particularly the high pre-treatment mortality and default from treatment. Further research aims to investigate these areas in more detail.

However, to date, there is a dearth of documented program experiences from resource-constrained settings treating co-infected patients; the global cohort of HIV/MDR-TB co-infected patients being treated is still discouragingly small.

Despite these limitations, and despite high fluoroquinolone resistance and extensive prior second-line treatment, this report provides encouraging evidence of the feasibility of offering ambulatory MDR-TB treatment in HIV-infected populations, even in a challenging, urban, resource-constrained setting. This community-based treatment model may have been successful in reducing mortality of HIV/MDR-TB co-infected patients. Rapid scale-up of both antiretroviral therapy and second line treatment for MDR-TB is needed to ensure survival of co-infected patients, and control the emerging epidemic of drug resistant TB in Mumbai.
